# Efficacy of intra-articular injection of allogeneic platelet-rich plasma for knee osteoarthritis combined with hematologic blood dyscrasias with platelet dysfunction: protocol of a randomized, double-blind, placebo-controlled trial

**DOI:** 10.1186/s12891-022-06073-3

**Published:** 2022-12-14

**Authors:** Xiaohang Zhu, Lingying Zhao, An Liu, Ziqiang Yu, Jiong Jiong Guo

**Affiliations:** 1 Department of Orthopedics and Sports Medicine, The First Affiliated Hospital of Soochow University, 188 Shizi Street, Suzhou, 215006 People’s Republic of China; 2 Department of Hematology, National Clinical Research Center for Hematologic Disease, The First Affiliated Hospital of Soochow University, 188 Shizi Street, Suzhou, 215006 People’s Republic of China; 3 Jiangsu Institute of Hematology, Key Laboratory of Thrombosis and Hemostasis of Ministry of Health of PR China, Suzhou, People’s Republic of China; 4 Suzhou Medical College of Soochow University, Suzhou, People’s Republic of China

**Keywords:** Allogeneic platelet-rich plasma, Knee osteoarthritis, Hematologic blood dyscrasias, Platelet dysfunction, Clinical trial

## Abstract

**Background:**

Autologous platelet-rich plasma (PRP) has been shown to alleviate the symptoms of patients suffering from knee osteoarthritis (KOA), but for certain patients with hematologic diseases with platelet dysfunction and patients receiving anti-platelet medications, autologous PRP is not an optimum solution. Allogeneic PRP has been proven to be safe and effective in the treatment of osteoarthritis, rotator cuff disease, refractory wounds and other medical fields. However, a well-designed and long-term follow-up prospective randomized controlled trial (RCT) to evaluate the effect of allogeneic PRP intra-articular injections for KOA combined with hematologic blood dyscrasias has not yet been performed.

**Methods/ design:**

We will conduct an allogeneic PRP injection for KOA combined with hematologic blood dyscrasias with platelet dysfunction study: a prospective, randomized, double-blind, placebo-controlled trial. One hundred participants with KOA combined with hematologic blood dyscrasias with platelet dysfunction will be randomly allocated to receive either one allogeneic PRP injection or one saline injection into the knee joint. The primary outcome will be a 12-month change in Western Ontario and McMaster Universities Osteoarthritis Index (WOMAC) score. Secondary outcomes will be the 36-Item Short-Form General Health Survey (SF-36) score, Lysholm score, overall knee pain score and MRI assessment at 1-, 3-, 6- and 12-month.

**Discussion:**

The results of this study will help determine whether allogeneic PRP could be used as a non-surgical intervention to treat patients with knee OA combined with hematologic blood dyscrasias with platelet dysfunction.

**Trial registration:**

Chinese Clinical Trials Registry reference: ChiCTR2100048624. Prospectively registered 11th of July 2021.

**Supplementary Information:**

The online version contains supplementary material available at 10.1186/s12891-022-06073-3.

## Background

Generally, knee osteoarthritis (KOA) is the most common cause of knee pain and dysfunction in the elderly. Osteoarthritis usually affects the medial compartment of the knee joint, which is related to misalignment. Currently, most research has focused on the treatment of pain relief and prevention of decline in function [1, 2].

Autologous platelet-rich plasma (PRP) is a safe autologous blood product containing high levels of growth factors and cytokines with potential to alter biological processes implicated in OA pathogenesis and symptoms [3]. In addition, several studies have reported that compared with non-steroidal anti-inflammatory drugs (NSAIDs), steroid and hyaluronic acid, PRP has favorable pain and function outcomes, [4–6] suggesting the potential benefits of PRP in treating KOA. Some alternative physical therapy, such as low-level laser therapy, needs to be further confirmed for efficacy in KOA patients [7].

Recent systematic reviews concluded that platelet dysfunction might have an impact on the efficacy of PRP [8]. For certain patients with hematological diseases (e.g. immune thrombocytopenia, aplastic anemia, systemic lupus erythematosus, etc.) and patients receiving anti-platelet medications, autologous PRP is not an optimum solution. Allogeneic platelet-rich plasma has been shown to be safe and effective in treating osteoarthritis, rotator cuff dysfunction, refractory wounds, and other medical fields [9–12]. Given the clinical effect on pain reduction in osteoarthritis and a good safety profile, allogeneic PRP is a promising nonsurgical therapy for knee OA combined with hematologic blood dyscrasias with platelet dysfunction. However, clinical trials of efficacy to date have been limited by a high risk of bias in allogeneic PRP trials. Although the results show that allogeneic PRP is safe and can relieve pain and recover articular function, the trials lack adequate blinding and control group suggesting the results are not convincing [11, 12]. Well-designed and long-term follow-up prospective randomized controlled trials (RCTs) have not yet been performed.

The aim of this study is to evaluate the safety and efficacy of intra-articular injection of allogeneic PRP from blood donors in patients with knee osteoarthritis combined with hematologic blood dyscrasias with platelet dysfunction. (e.g. immune thrombocytopenia, aplastic anemia, etc.) Our hypothesis is that there is a clinically relevant difference in the primary and secondary outcomes between the two treatment groups. The minimal clinically important differences of patient reported outcomes will be considered in this study.

## Method/design

### Trial design

The allogeneic PRP injection for knee OA study is a prospective, randomized, double-blind, placebo-controlled trial design. This study protocol follows the recommendations of the SPIRIT Statement (supplement 1) [13] for clinical trial protocols elaboration and is reported according to the CONSORT Statement (supplement 2) [14]. The trial will be conducted at The First Affiliated Hospital of Soochow University, which is one of the three national clinical research centers for hematologic disease in China. The primary endpoint for the analysis of outcomes is 12 months after baseline assessment. The trial is registered at the Chinese Clinical Trial Registry with a number of ChiCTR2100048624 and follows the World Health Organization’s guidelines.

### Participants

We will recruit 100 participants with painful knee OA combined with hematologic blood dyscrasias with platelet dysfunction from outpatient department. Participants are eligible for the study if they meet all inclusion criteria below:

i. age between 18 and 80 years
ii. unilateral symptomatic knee with history of chronic pain (at least 4 months) or swelling
iii. hematologic blood dyscrasias with platelet dysfunction
iv. limitation of daily activities
v. radiographic imaging findings of knee osteoarthritis (Kellgren and Lawrence grade 1–3)

The exclusion criteria are as follows:

i. systemic cardiovascular or neurologic disorders, diabetes septicemia or fever
ii. cutaneous infections in the area to be injected
iii. had injection into the target knee joint of glucocorticoid in the past 3 months or hyaluronic acid in the past 6 months
iv. use of nonsteroidal anti-inflammatory drugs (NSAIDs) within 5 days before treatment
v. previous knee surgery
vi. have systemic or inflammatory joint disease such as rheumatoid arthritis
vii. have other muscular or joint affecting lower limb function

### Procedures

**Fig. 1:**
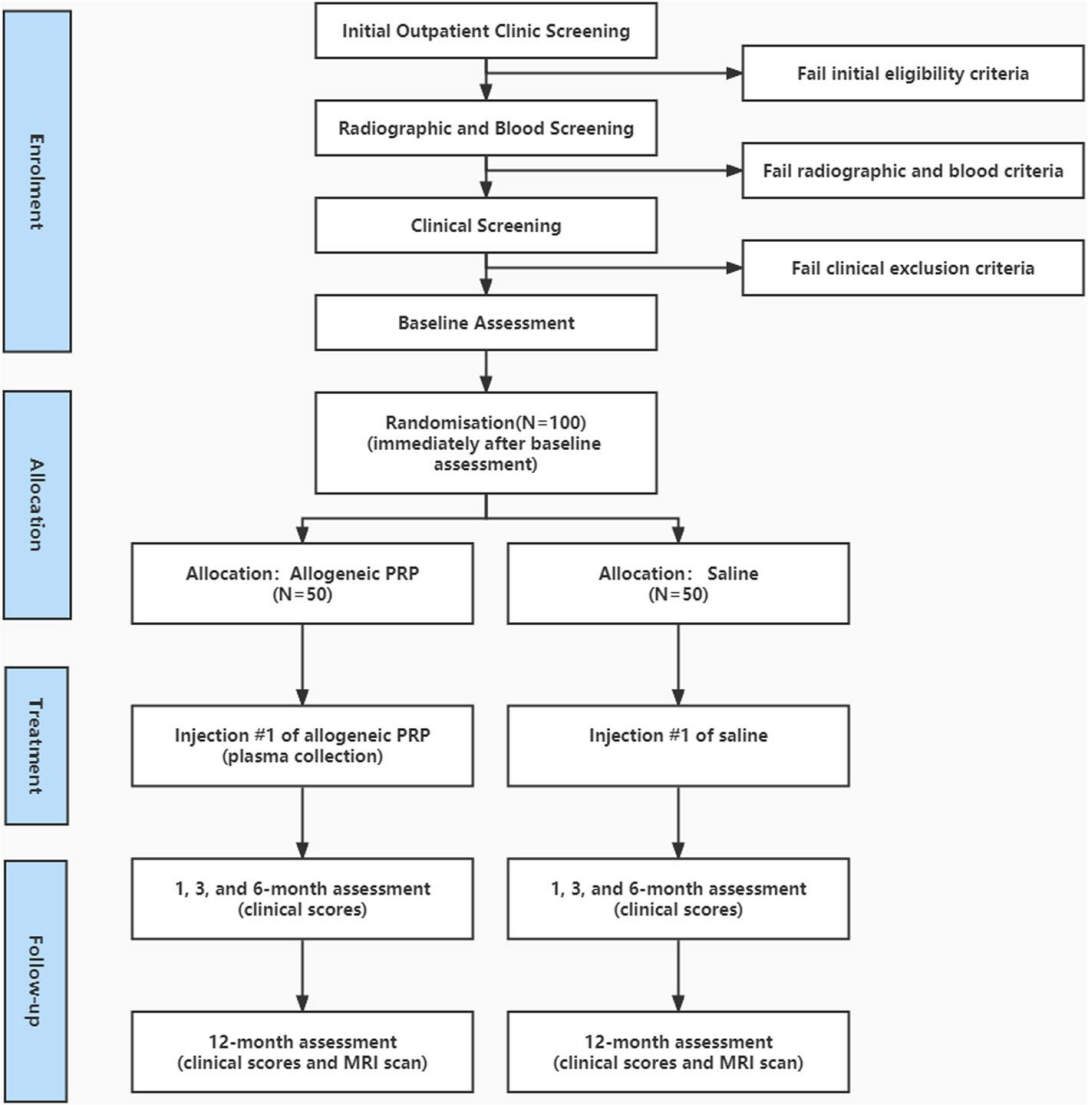
Flow diagram of the study protocol

Figure 1 and Table 1 outline the trial phases and the schedule for enrollment, screening, interventions and assessments. Participants will complete an onsite survey in the outpatient department to determine initial qualifications, and then a coordinating researcher will screen through a questionnaire. Participants deemed appropriate from the questionnaire screening will be allowed to undergo conventional radiographs (weight-bearing long-leg, A/P and lateral views) and blood screening. The screening will be performed in the outpatient department, where two experienced physicians evaluated patients’ eligibility for enrollment in this study through history taking, imaging examination, and laboratory tests. These steps could diminish possible bias about the inclusion criteria of age range. Participants who meet the inclusion and exclusion criteria will then be deemed suitable for the study. Eligible participants will complete the baseline clinical outcome scores, paper-based questionnaires and baseline knee MRI scans. Participants will also be asked to discontinue non-steroidal anti-inflammatory drugs and other analgesics for knee pain from 3 weeks before the baseline assessment until the 12-month follow-up assessment.

**Table 1:** Schedule of enrolment, interventions and assessments

Time point			Study period				
	Enrolment	Baseline	Injection	Post-allocation			Close-out
			1	1 M	3 M	6 M	12 M
**Enrolment**							
**Eligibility screen**	**×**						
**Informed consent**	**×**						
**Allocation**		**×**					
**Interventions:**							
**Platelet rich plasma**			**×**				
**Saline**			**×**				
**Assessments:**							
**Primary outcomes**							
^**a**^**WOMAC score**		**×**		**×**	**×**	**×**	**×**
**Secondary outcomes**							
^**b**^**VAS**		**×**		**×**	**×**	**×**	**×**
**Lysholm score**		**×**		**×**	**×**	**×**	**×**
^**c**^**SF-36**		**×**		**×**	**×**	**×**	**×**
**MRI analysis**		**×**					**×**
**Other measures**							
**Drug/supplement use**		**×**		**×**	**×**	**×**	**×**
**Platelets and leukocytes counts, VEGF and TGF-β**			**×**				
**Number of injections**			**×**				
**Adverse events**			**×**	**×**	**×**	**×**	**×**
**Success of blinding**			**×**				
**Descriptive measures**							
**Weight, height, BMI, Kellgren and Lawrence grade, Age, gender, duration of symptoms**		**×**					
**Injection site, Complications**			**×**				

^a^WOMAC score, The Western Ontario and McMaster Universities Osteoarthritis Index; ^b^VAS, visual analogue scale; ^c^SF-36, the short-form 36 item health survey questionnaire

Paper-based clinical outcome scores will be distributed to participants at 1-, 3-, 6-, and 12-month to complete primary and secondary outcomes at the outpatient department. Knee MRI scans will be conducted at 12-month at the radiology center of the First Affiliated Hospital of Soochow University. At the last follow-up, patients will be asked if they would like to receive allogeneic PRP injection again. This will be registered accordingly.

### Monitoring committee

The trial will be monitored by the clinical research monitoring committee of the First Affiliated Hospital of Soochow University. The monitoring committee aims to improve the quality of clinical research and ultimately, patient care. Before the trial commenced, a monitoring plan was set up. Monitors will be required to verify that:

1. the conduct of the trial is compliant with the currently approved protocol and with applicable laws regulatory requirements, for example, WMO, GCP (China), ISO14155 and laws on clinical research in China;
2. the rights and privacy of patient subjects are protected;
3. The research data is accurate, complete, and true.

Monitoring will be performed at the initiation of the study until the results of the study are published.

### Randomization procedures

Randomization is centralized, and participants will be randomly allocated in a 1:1 ratio. An independent statistician will provide a computer-generated randomization list, using a permuted block design with block sizes of 4 and 6. The sequence is concealed by the use of a computer interface implemented in the electronic case report form. The patients, treating physicians and coordinating researchers will all be blinded to the allocation of the intervention. A study nurse will have access to the randomization result and the allocated intervention.

### Blinding arrangements

Participants will not be told until the end of the study which group they were allocated to. The study nurse will prepare the injection in a separate room and place an opaque label around the syringe and needle base to mask the contents. The nurse will then give the syringe to the injecting treating physician who will not know or be able to tell whether the syringe has allogeneic PRP or saline. All clinical and MRI assessments will be conducted by two radiologists with over 10 years of clinical work experience. All evaluators are blinded to treatment allocation.

### Interventions

Participants in both the allogeneic PRP and saline groups will receive one intra-articular knee injection at the same time after signing written informed consent (supplement 3), in line with commonly used injection protocols for this condition [6, 15]. The study nurse will give the treating physician an occluded syringe with a needle containing 4 ml of either normal saline or allogeneic PRP, and the contents will be injected into the knee joint. After the injection, passive knee flexion and extension will be performed six times with the participant observed resting for 20 min thereafter.

After injection, participants will be asked which group they believe they are in, and separately the injecting treating physician will also be asked which group they believe the participant is in to assess the success of blinding. This will be registered accordingly. The study nurse will ask the participant if they experienced any adverse events following the injection.

### PRP preparation

One healthy volunteer donor will donate about 300–400 ml (according to the definite number of participants) of peripheral venous blood. To assess the safety of the allogeneic PRP, tests for hepatitis B, hepatitis C, human immunodeficiency virus, and syphilis will be performed and confirmed before application. Every 8 ml of venous blood for each participant (as recommended by the company) is drawn manually using butterfly safety locks and emptied into 10-ml standard tubes (Regenlab PRP Kit-RegenACR®, Le Mont-sur-Lausanne, Switzerland) and are then put in RegenLab PRP Centrifuges. The centrifugation settings are set at 1500 g for 5 minutes. After centrifugation, the blood is fractionated. By gently inverting the Regen BCT tubes several times and process for resuspension of the cellular deposit in the supernatant, approximately 4 ml of PRP will be obtained from each tube. The PRP used for injection will not be activated before application. About 4 ml of PRP will be injected for each patient in the PRP group. In addition, one tube of PRP will be sent to the laboratory for platelet and leukocyte counting, and growth factor (VEGF and TGF-β) analysis. The whole process will be carried out by a study nurse in a separate room and the syringe and needle base will be placed an opaque label to mask the contents.

### Saline injections

The study nurse will prepare a syringe with a saline solution (4 mL), attach a needle and place an opaque label around the syringe and needle base to mask the contents in a separate room. The nurse will then deliver the syringe to the treating physician for injection.

### Outcome measures

Study outcome measures are presented in Table 1. The primary endpoint measure are 12-month change in knee pain and function scores.

i. Knee pain and function score: this will be measured at baseline, 1-, 3-, 6- and 12-month using the Western Ontario and McMaster Universities (WOMAC) Osteoarthritis Index with terminal descriptors ‘mild OA (score <32)’, ‘moderate OA (score 32~48)’ and ‘severe OA (score >48)’. This was chosen because it has well-established clinimetric properties in knee OA and established minimal clinically important differences, and is a recommended measure for knee OA RCTs from the Osteoarthritis Research Society International [16–18].

The following secondary outcome measures will also be collected:

i. Health-related quality of life: this will be measured at baseline, 1-, 3-, 6- and 12-month using the short-form 36-item health survey questionnaire (SF-36) instrument [19, 20]. The SF-36 is divided into 8 fields, with higher scores indicating better quality of life.
ii. Overall knee pain score during activities of daily living (using an 11-point VAS): this will be measured at baseline, 1-, 3-, 6- and 12-month. The VAS is to indicate the degree of pain in a total of 11 numbers from 0 to 10; 0 means no pain and 10 means worst pain with high reliability. The patient chooses a number to represent the degree of pain according to his pain.
iii. Lysholm knee scoring scale: this will be measured at baseline, 1-, 3-, 6- and 12-month. The Lysholm knee scale demonstrated overall acceptable psychometric performance for outcomes assessment of various chondral disorders of the knee. This makes up for the shortcomings of the WOMAC score and established minimal clinically important differences with high reliability [17, 21].
iv. MRI-derived measurements: an MRI scan of the target knee will be performed at baseline and 12-month using a 3 T whole-body system with a dedicated extremity coil and a T1-weighted fat-suppressed 3D gradient recall acquisition sequence.

MRI osteoarthritis knee score (MOAKS): an OA-specific semi-quantitative tool evaluating multi-feature joint changes associated with OA [22]. We will assess the following subscores:

Meniscal morphology: any region worsening at 12-month; scored as yes or no.

Intercondylar synovitis incorporating synovitis and effusion: worsening at 12-month; scored as yes or no.

Whole knee effusion: categorized as worsened, no change, or improved.

Progression of medial distal femur and proximal tibia bone marrow lesion size: scored as 0–3 per region, with higher scores indicating greater size.

Cartilage defects: score in the medial distal femur and the proximal tibia at baseline and 12-month using categorical scoring (range 0–4 per region, with higher scores indicating greater cartilage defects).

Progression of medial cartilage defects (yes/no) will be defined as an increase in score by at least 1 from baseline to follow-up in either the medial tibial or medial femoral compartment [23].

### Co-intervention

Participants will be asked to avoid the use of non-steroidal anti-inflammatory drugs and other analgesics for knee pain from 3 weeks before the baseline assessment until the 12-month follow-up assessment. Throughout the trial, key treatment (exercise therapy and healthy lifestyle advice) and co-interventions used by patients will be registered, such as non-steroidal anti-inflammatory drugs, other analgesic drugs, intra-articular injections or inlays.

### Safety

All adverse events are reported spontaneously by the patient or observed by the study nurse. This will include questions about any adverse events that participants believe may be related to the trial intervention, including their nature, how long they lasted for and what action if any, they took. To date, no serious adverse events have been reported in the literature concerning PRP intra-articular injections of the knee. The local Medical Ethical Commission will be notified of any serious adverse events. In the event this happens, the advice of the Medical Ethical Commission will be followed accordingly.

### Loss to follow-up

The coordinating researchers will attempt to reduce loss to follow-up as much as possible by contacting every patient and being present at every patient visit. Each patient will be assisted by a coordinating researcher to ensure that each paper-based questionnaire is filled in. If the patient is lost to follow-up, an analysis of demographic and prognostic characteristics will be performed on these cases and the remaining patients. The patient eligible for and compliant with each follow-up will be documented.

### Missing items

If the proportion of missing data exceeds 10%, missing outcome data will be imputed using multiple imputation methodology and the sensitivity of the missing at random assumption will be investigated [24].

### Sample size calculations

The sample size calculation is based on the primary outcome measure, the WOMAC score at 12-month. Based on previous studies, [5, 16, 17, 25] a 11.5 points reduction in WOMAC total score was considered as the minimum difference to be detected between the groups with a standard deviation (SD) of 16. Accepting a false-positive rate of 5% (α = 0.05) and a power of at least 90% (β = 0.10), we determined a theoretical minimum sample size of 84 patients. Considering the estimated 15% dropout rate, a total of 50 patients per group were required. Patient recruitment will be terminated in both groups when the required minimum number of patients is attained.

### Data management

Paper-based questionnaires will be the primary data collection tools for the study. The questionnaires will be completed at the outpatient clinic at baseline and each follow-up time point. On receipt of the questionnaire forms, the coordinating researchers will make a visual check of the responses and query missing data when possible. The paper-based questionnaire will be securely kept by a study nurse.

A study nurse, blinded to the group allocation, will store the forms into an electronic database by double data entry to minimise typing errors. The coordinating researchers, blinded to the group allocation, will perform a visual check of the data in the electronic database and then query all missing, implausible and inconsistent data. Patient records in the participating hospitals will be used when collecting missing data or interpreting inconsistent or implausible data.

After 12-month follow-up visits are completed and all data stored, a research assistant, not involved in the trial, will aggregate all the data into a separate database, which will be the source for the final data analysis. Participant files will be maintained in storage (both in electronic and paper formats) at the coordinating center for a period of 10 years after completion of the study (10-year follow-up visits).

### Statistical analysis

We will conduct an intention-to-treat analysis whereby all participants will be included in the study in the group to which they were randomized. The analysis will be conducted by a statistical expert blinded to the trial, with two-sided hypothesis tests and *p*-values < 0.05 significant.

Baseline characteristics will be analyzed between groups using descriptive statistics. Continuous variables will be expressed in terms of the means and the standard deviations, and compared using the student t test (normally distributed) or Mann-Whitney U test (nonnormally distributed). The categorical data will be expressed as frequency and compared using the Chi-square test or Fisher’s exact test. Normality will be confirmed using the Kolmogorov-Smirnov test.

### Primary outcomes analysis

To test for the effect of treatment on the between-group difference in the primary outcome (WOMAC score), we will use a repeated measurement general linear model (GLM) with Sidak test for multiple comparisons. Changes from baseline to all follow-up time points (1-, 3-, 6- and 12-month) will be included in the model to assess the differences in the primary outcome at different follow-up times. The GLM will also be used as a multivariate analysis to assess the influence of the treatment on the follow-up evolution of all the clinical scores and objective measures performed. The mixed linear regression model (MLM) will be used to assess whether PRP effects on the primary outcome at 12 months will be moderated by age, body mass index, and Kellgren and Lawrence grade. The between-group differences of continuous, normally distributed and homoscedastic data will be compared for their means using the analysis of variance (ANOVA). Nonnormal data will be compared using the Mann-Whitney U test.

### Secondary outcomes analysis

To test for the effect of treatment on between-group differences in secondary outcomes, we will also use the repeated measurement general linear model (GLM) with the Sidak test for multiple comparisons. Changes from baseline to all follow-up time points will also be included in the model. The statistical analysis method is similar to the primary analysis. MRI-derived measurements will be compared between groups using appropriate models, adjusting for age, sex, body mass index, and the stratifying variables. The success of blinding will be assessed using the James Blinding Index [26].

### Ethics and dissemination

The trial will be conducted according to the principles of the Declaration of Helsinki. Ethical approval has been obtained from the National Clinical Research Center for Hematologic Disease, the First Affiliated Hospital of Soochow University Ethics Committee (No.2021–356). The trial has been registered in the Chinese Clinical Trial Registry (ChiCTR2100048624) and any revisions about the protocol are documented in this registry. All participants will provide written informed consent before any study-related procedures.

The results of the trial will be reported first to trial collaborators. The main report will be drafted by the trial coordinating team, and the final version will be agreed upon by the Trial Steering Committee before submission for publication, on behalf of the collaboration.

The results of this trial will substantially inform clinical practice on the clinical effectiveness and safety of the treatment in these patients. The results of this project will be disseminated through peer-reviewed journals, conference presentations, the National Library for Health and through local mechanisms at the participating center.

## Discussion

This is a prospective, randomized, double-blind, placebo-controlled trial evaluating the efficacy and safety of intra-articular injection of allogeneic PRP from blood donors in patients with knee OA combined with hematologic blood dyscrasias with platelet dysfunction who are unsuitable for autologous PRP therapy. The primary outcome is the WOMAC score at 12-month. Our study will also evaluate the long-term efficacy of allogeneic PRP for up to 5 years.

Autologous PRP has been shown to alleviate the symptoms of patients suffering from knee OA and allogeneic PRP has been proven to be safe and effective in the treatment of osteoarthritis, rotator cuff disease, refractory wounds and other medical fields [3, 9–12]. In the absence of effective evidence-based non-surgical interventions for patients with knee OA combined with hematologic diseases with platelet dysfunction, allogeneic PRP would fulfill a clear clinical need. There was only one preliminary study on the efficacy of allogeneic PRP for knee OA with short follow-up periods, no control group, and low evidence [12]. In addition, all PRPs used for injection in the present study are from the same healthy donor at the same time and prepared with the same system. This avoids confounding factors and high risk of bias.

A positive study outcome is that allogeneic PRP could be used as a non-surgical intervention to treat patients with knee OA who are unsuitable for autologous PRP therapy. A negative outcome will prevent the widespread use of a non-efficacious treatment.

This study has potential limitations. First, the generalizability of results to other PRP products and treatment regimens remains unknown. All PRP products differ in their production method and content, and different treatment regimens regarding dose, timing and number of injections are used in clinical practice. However, we prepare PRP using commercial kits by standardized procedures and analyzed related components. Second, this study will not control for variations in physical therapies between the two groups. Throughout the trial, the exercise therapy and lifestyle will be registered. Third, the population recruited is from a single center. However, as our center is one of the three national clinical research centers for hematologic disease in China, it will help us screen a homogenous group of participants better.

## Supplementary Information

**Additional file 1: Supplement 1.** SPIRIT Checklist.

**Additional file 2: Supplement 2.** CONSORT Checklist.

**Additional file 3: Supplement 3.** Patient Consent Form.

## Data Availability

All data will be used only for analysis of the present study and will be protected from any unnecessary exposure. All information will be published confidentially, without the name of the subjects exposed. All data will be available for review and confirmation of data analysis when requested by a review process for publication of the article in indexed scientific journals or presentations at scientific events.

## Abbreviations

SPIRIT: Standard Protocol Items: Recommendations for Interventional Trials
CONSORT: Consolidated Standards of Reporting Trials
WOMAC score: The Western Ontario and McMaster Universities Osteoarthritis Index
VAS: Visual analogue scale
SF-36: The short-form 36 item health survey questionnaire
MOAKS: Magnetic resonance imaging Osteoarthritis Knee Score
MRI: Magnetic resonance imaging
KOA: Knee Osteoarthritis
RCT: Randomised controlled trial
