# Supplementary material for: Efficacy of intra-articular injection of allogeneic platelet-rich plasma for knee osteoarthritis combined with hematologic blood dyscrasias with platelet dysfunction: protocol of a randomized, double-blind, placebo-controlled trial
Source: BMC Musculoskelet Disord. 2022 Dec 14;23:1095. doi: 10.1186/s12891-022-06073-3 (PMC9749297; doi:10.1186/s12891-022-06073-3)
Supplement: Supplementary file 3 — Additional file 3: Supplement 3. Patient Consent Form. [file 12891_2022_6073_MOESM3_ESM.docx]

PATIENT CONSENT FORM

We would like to invite you to take part in our research study, investigating the benefit of a new type of treatment for knee osteoarthritis combined with hematologic blood dyscrasias with platelet dysfunction.

Before you decide whether to take part we would like you to understand why the research is being done and what it would involve for you. A researcher from our team will go through this information sheet with you and answer any questions you have.

**KEY CONTACTS**

Dr. Guo JJ (Professor of soochow University, doctor of the First Affiliated Hospital of Soochow University)

Tel: 0512 6797 2128

Email: [drjjguo@163.com](javascript:;)

The First Affiliated Hospital of Soochow University: <http://fyy.sdfyy.cn>

Soochow University: <https://www.suda.edu.cn>

1. **Background**

You have been diagnosed with knee osteoarthritis combined with hematologic blood dyscrasias with platelet dysfunction. Autologous PRP has been shown to alleviate the symptoms of patients suffering from knee osteoarthritis, but for certain patients with hematologic diseases with platelet dysfunction and patients receiving anti-platelet medications, autologous PRP is not an optimum solution. Allogeneic PRP has been proven to be safe and effective in the treatment of osteoarthritis, rotator cuff disease, refractory wounds and other medical fields. However, a well-designed and long-term follow-up prospective RCT to evaluate the effect of allogeneic PRP intra-articular injections for KOA combined with hematologic blood dyscrasias has not yet been performed.

1. What is the purpose of this study?

The objective of this study was to evaluate the safety and efficacy of intra-articular injection of allogeneic PRP from blood donors in patients with knee osteoarthritis combined with hematologic blood dyscrasias with platelet dysfunction. We would like to look into this further with your help. The study is funded by National Clinical Research Center for Orthopedics, Sports Medicine & Rehabilitation and is coordinated by Soochow University. Dr Guo JJ is the overall lead for this study.

This new treatment involves taking a small sample of donors’ blood, mixed with anticoagulant, to stop the blood clotting, which is then spun in a machine to separate out the components of the blood. The part of the blood we are interested in is the plasma containing a high number of platelets, known as platelet rich plasma.

Autologous PRP play an important role in the treatment processes within KOA. The clinical trial plans to test whether allogeneic PRP injection help with KOA combined with hematologic blood dyscrasias with platelet dysfunction. By injecting the allogeneic PRP into the Knee, you may experience increased healing and reduced pain.

If you participate in the study, you will be asked to provide information about your pain, ability to perform activities, complications and overall health. The study is important as we want to see whether this treatment is the best for future patients with KOA combined with hematologic blood dyscrasias with platelet dysfunction so that they will receive the best possible treatment.

1. Why have I been chosen?

You have been chosen because you have had KOA combined with hematologic blood dyscrasias with platelet dysfunction for more than 4 months. There will be the First Affiliated Hospital of Soochow University taking part in the study and 100 patients will take part in total.

1. Do I have to take part?

It is up to you whether or not to take part. If you decide to take part you will be given this information sheet and asked to take as much time as is required to consider your decision. If you decide to proceed you will sign a consent form. If you decide to take part you are still free to withdraw at any time and without giving a reason. A decision to withdraw at any time or a decision not to take part will not affect the standard of care you receive.

1. Which treatment will I receive?

You will be allocated to either the platelet rich plasma (PRP) injection or the placebo injection. The allocation process will be done by a computer and is done purely by chance. There is an equal chance of you receiving either the PRP injection or the placebo injection. We need to have a placebo injection in order to clearly see whether the PRP injection treatment is the best to use for future patients with KOA combined with hematologic blood dyscrasias with platelet dysfunction. You will not be told which treatment you are allocated to. Twelve months after treatment, when you receive your 12-month follow up questionnaire, you will be asked if you would like to know the allocation you received. If you indicate that you would, then the trial team will contact you to let you know.

1. What will happen if I take part?

Enrollment: Questionnaires and basline assessment

You will be asked to complete a questionnaire (baseline questionnaires) about your pain, activity and current health and attend the radiology centres for their baseline knee MRI scans. You will not be told which treatment you will be allocated but an independent research assistant will know and allocate the treatment.

Interventions: If you are allocated to receive the PRP injection treatment, allogeneic PRP sample will be injected into the knee. If you are allocated to receive the placebo injection, saline will be injected into your knee. You will undergo one intra-articular knee injection. You will receive a care and information sheet following the injection procedure. You will receive a standard recovery programme by your clinician and asked not to do any other treatments for 12 months. This makes sure everyone in the study receives similar treatment after the injection.

Post-injection treatment schedule of events:

1-, 3-, 6-month: You will receive a paper-based questionnaires in the post asking about your current pain, activity and current health so we may have an update on your condition in the outpatient clinic. This will only take 30min of your time.

12-month: You will be ask to complete a paper-based questionnaires in the post asking about your current pain, activity and current health so we may have an update on your condition in the outpatient clinic. This will only take 30min of your time. Then you will receive MRI scans at radiology center of the First Affiliated Hospital of Soochow University.

It is really important that we receive your completed questionnaires as the answers you provide will give us an indication of how effective the treatment you have been given.

We will ask you for your name, address, telephone numbers and next of kin contact. Next of kin details will be used in the event that we are unable to reach you through the contact details provided. Please ensure that you notify your next of kin that you have shared their contact information with the the First Affiliated Hospital of Soochow University Clinical Trials Unit. All information will be treated with the strictest security and confidentiality.

1. What are the possible disadvantages and risks of taking part?

There are no specific risks of receiving allogeneic PRP because it has been proved to be safe and successfully used in the treatment of many medical fields. However disadvantages of receiving any injection include soreness, bruising and swelling at the injection sites, this is a common effect. There is also a very low risk of infection, but this is no greater than when receiving any injection. We are not aware of any risks over and above those when receiving any injection.

1. What are the possible benefits of taking part?

We do not know whether this new treatment will give the best results therefore there may be no immediate benefit to you for taking part. You may receive the PRP injection which may aid your treatment of KOA. There are no known risks to receiving PRP. The information that you provide us with by taking part in the trial may inform us about future treatments.

1. What if new information becomes available?

Sometimes during the course of a research project, new information becomes available about the treatment that is being studied. If this happens, a researcher from the First Affiliated Hospital of Soochow University will tell you about it and discuss with you whether you want to continue in the study. If you decide to withdraw, we will encourage you to discuss your continued treatments with your doctor. If you decide to continue in the study you will be asked to sign an updated consent form.

1. What happens when the research study ends?

You will be in the study for 12 months. If you are still having problems after this time, your clinician will arrange for you to have an appointment with an appropriate specialist to continue your treatments.

1. What happens if there is a problem?

In the unlikely event that something does go wrong and you are harmed during the research and this is due to someone‘s negligence then you may have grounds for a legal action for compensation against the First Affiliated Hospital of Soochow University. This study is covered by the the First Affiliated Hospital of Soochow University’s insurance and indemnity cover. If you have an issue, please contact the Chief Investigator of the study: [drjjguo@163.com](javascript:;).

1. Who should I contact if I wish to make a complaint?

Any complaint about the way you have been dealt with during the study or any possible harm you might have suffered will be addressed. Please address your complaint to the person below, who is a Senior the First Affiliated Hospital of Soochow University Official, entirely independent of this study: Telephone: 0512 6797 2000

1. Will my taking part in this study be kept confidential?

All information which is collected about you, and your next of kin, during the course of the research will be kept strictly confidential. Any information about you which leaves the First Affiliated Hospital of Soochow University will have your name and address removed so that you cannot be recognised from it. The attached trial Data Transparency Statement provides further detail around how all sensitive and confidential information will be handled.

1. What will happen to the results of the research study?

The study is expected to be completed by 31st January 2024. Once all of the data have been gathered, we will publish the findings in medical journals and at medical conferences. You will not be identified in any reports or publications resulting from the study. If you would like to know the final results of the study we will ask you to indicate this on your 12-month follow up questionnaire. If you do, we will post you a summary of the study results, once the study is complete.

1. What will happen if I decide not to participate in the research study?

If you decide not to participate in the research study your treatments will not be affected. You are free to withdraw consent from the trial at any time.

1. Who has reviewed this study?

This study has been reviewed by the First Affiliated Hospital of Soochow University Ethics Committee.

17. Contacts for further information

If, at any time, you would like further information about this research project you may contact your clinician, telephone number 0512 6797 2134

Whether to participate in this trial(YES OR NO):______

Name:_______ Date:_______

(If you are unable to complete the signature, it will be signed by your next of kin )
